# Desmoplakin cardiomyopathy—an inherited cardiomyopathy presenting with recurrent episodes of acute myocardial injury

**DOI:** 10.1007/s12471-022-01735-2

**Published:** 2022-11-26

**Authors:** S. A. C. Schoonvelde, A. Hirsch, S. C. Yap, J. M. A. Verhagen, M. A. van Slegtenhorst, D. Segers, J. E. van Loon, M. Michels

**Affiliations:** 1 Department of Cardiology, Erasmus Medical Centre, University Medical Centre Rotterdam, Rotterdam, The Netherlands; 2 Department of Radiology and Nuclear Medicine, Erasmus Medical Centre, University Medical Centre Rotterdam, Rotterdam, The Netherlands; 3 Department of Clinical Genetics, Erasmus Medical Centre Rotterdam, University Medical Centre Rotterdam, Rotterdam, The Netherlands; 4 Department of Cardiology, Amphia Hospital, Breda, The Netherlands; 5 Department of Cardiology, Haga Hospital, The Hague, The Netherlands

**Keywords:** Desmoplakin, Inherited cardiomyopathy, Non-infectious myocarditis, Recurrent cardiac injury, MINOCA

## Abstract

We present two female patients with recurrent episodes of myocardial injury, consisting of acute chest pain and elevated cardiac markers without coronary artery disease. Cardiovascular magnetic resonance imaging identified extensive late gadolinium enhancement suggestive of an inherited cardiomyopathy. Genetic testing showed heterozygous pathogenic variants in the desmoplakin (*DSP) *gene, the gene coding for the desmoplakin protein, a structural protein found in the cardiac desmosome. Pathogenic variants in the *DSP* gene are associated with dilated and arrhythmogenic cardiomyopathy. *DSP* cardiomyopathies may cause recurring myocardial injury mimicking an acute coronary syndrome or myocarditis. Cardiac magnetic resonance imaging is key in its diagnosis due to its specifying imaging features. Genetic testing is essential for the evaluation and confirmation of the diagnosis.

**Video online:**

The online version of this article contains 2 videos. The article and the videos are online available (10.1007/s12471-022-01735-2). The videos can be found in the article back matter as “Electronic Supplementary Material”.

## Introduction

Inherited cardiomyopathies are diagnosed more frequently due to improved screening and diagnostic methodologies. The prevalence of inherited or idiopathic dilated cardiomyopathies (DCM), based on adult screening studies, is estimated to be approximately 1 in 250 [1]. Arrhythmogenic cardiomyopathies (ACM), being rarer, are estimated to occur in 1 to 2000 to 5000 adults. However, these estimates are limited by their methodology and the often incomplete phenotypical penetrance of underlying pathogenic DNA variants.

We describe two patients who presented with recurrent episodes of acute chest pain and elevated cardiac markers without coronary artery disease, in whom deep phenotyping and genotyping led to the diagnosis of a desmoplakin (*DSP*) cardiomyopathy. We discuss and review the current knowledge on *DSP *cardiomyopathy.

## Case reports

**Fig. 1:**
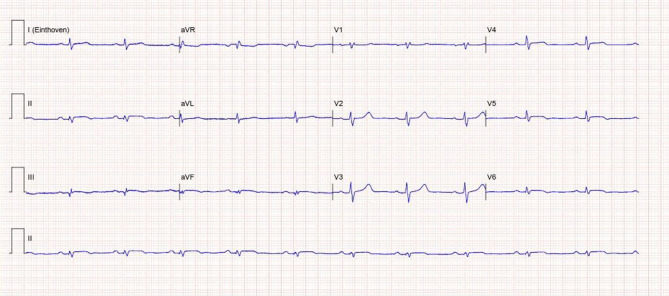
12-Lead electrocardiogram made at admission of patient A showing a sinus rhythm of 70 beats per minute, normal heart axis, normal conduction times, low voltage QRS complexes, and subtle inferolateral ST-segment elevations without reciprocal ST depressions

**Fig. 2:**
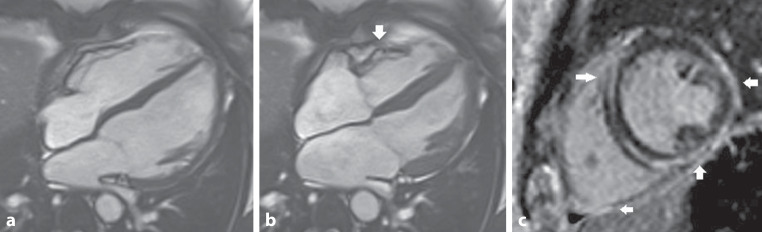
Cardiovascular magnetic resonance of patient A: **a**,**b** show the 4‑chamber end-diastolic (**a**) and end-systolic (**b**) balanced steady-state free precession cine images. Note the involvement of the right ventricle (*arrow*, **b**) with regional basal free wall dyskinesia. **c** shows extensive late gadolinium enhancement (LGE) on the short-axis assessment with involvement of the left and right ventricle. Epicardial LGE (*arrows*, **c**) is seen in the lateral and inferior left ventricular segments, and also mid-myocardial LGE in the septum and the right ventricle free wall. A cine movie is available (Video S1 in Electronic Supplementary Material)

**Patient A **is a 35-year-old woman who presented with acute chest pain and elevated cardiac markers (creatinine kinase [CK] 1991 U/l [upper limit of normal (ULN) 145] and high-sensitivity cardiac troponin T [hs-cTnT] 5510 ng/l [ULN 14]). Her medical and family history was unremarkable. The electrocardiogram (ECG) showed low-voltage QRS complexes and subtle inferolateral ST-segment elevations (Fig. 1). Transthoracic echocardiography showed a mildly reduced left ventricular systolic function with apical hypokinesia. Coronary angiogram excluded coronary artery disease. Cardiovascular magnetic resonance imaging (CMR) showed extensive epicardial late gadolinium enhancement (LGE) in the inferior and lateral wall and mid-myocardial LGE in the septum with preserved systolic left ventricular function (See Fig. 2 and Video S1 in Electronic Supplementary Material [ESM]). The patient was diagnosed with acute myocarditis and was discharged from the hospital a few days later.

During follow-up, symptomatic polymorphic premature ventricular contractions (2% burden during Holter monitoring) were present, successfully treated with atenolol. Approximately 20 months later, patient presented again with acute chest pain and elevated cardiac markers (CK 499 U/l and hs-cTnT 1300 ng/l) after intense physical exertion. Repeated CMR showed similar results. An ^18^F‑FDG positron emission tomography/computed tomography scan was normal, ruling out active sarcoidosis or myocarditis. The patient was referred to a tertiary centre for further analysis, including genetic testing. The CMR scans were reassessed, in hindsight showing an LGE pattern and regional right ventricular dyskinesia of the basal free wall with localised LGE suggestive for an inherited cardiomyopathy (Fig. 2). Genetic analysis was performed and a pathogenic variant (class 5) c.2297+2T > A in the *DSP* gene (NM_004415.2) with potential RNA splicing effect was found. Variants were classified according to the American College of Medical Genetics and Genomics (ACMG) guidelines [2]. An implantable loop recorder (ILR) was implanted. So far, no arrhythmias have occurred and the patient remains asymptomatic. Results of cascade genetic testing in first-degree relatives are pending.

**Fig. 3:**
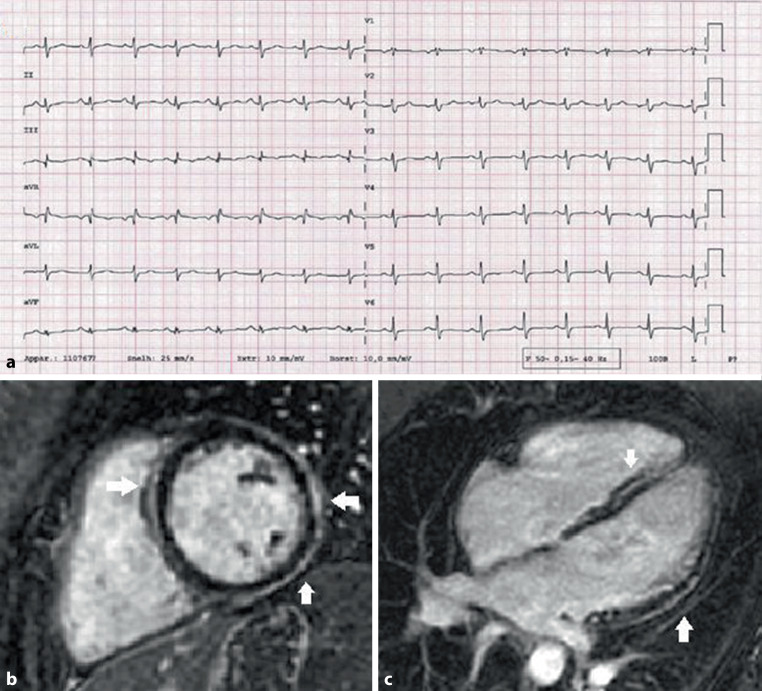
12-Lead electrocardiogram (**a**) made at admission of patient B showing a sinus rhythm of 95 beats per minute with a normal heart axis, normal conduction times, poor R‑wave progression, non-specific repolarization abnormalities and small QRS voltages. Cardiovascular magnetic resonance of patient B: **b** shows extensive ring-like late gadolinium enhancement (LGE) on the short-axial view. **c** shows the LGE in the 4‑chamber view within the septum and the lateral wall of the left ventricle. A cine movie is available (Video S2 in Electronic Supplementary Material)

**Patient B** is a 28-year-old woman with a history of type 1 diabetes mellitus and a family history of DCM with an affected father. She presented with acute chest pain and elevated hs-cTnT (831 ng/l) with normal CK values. Her ECG at presentation showed low-voltage QRS complexes and non-specific repolarisation abnormalities, particularly of the inferolateral leads, with no acute findings (Fig. 3). Echocardiography showed a mildly increased left ventricular end-diastolic diameter (56 mm) and a low-normal left ventricular ejection fraction (LVEF 50%). Coronary angiogram showed no coronary artery abnormalities. She was diagnosed with a myocardial infarction with non-obstructive coronary arteries (MINOCA). Patient received dual antiplatelet therapy, a statin and a non-dihydropyridine calcium channel blocker. CMR showed a pattern correlating with a non-ischaemic DCM with reduced LVEF (44%) and extensive epicardial LGE, most suggestive for an inherited cardiomyopathy (see Fig. 3 and Video S2 in ESM). She was referred to a tertiary centre for further analysis.

A few days after the CMR she presented again with acute chest pain and hs-cTnT increase (585 ng/l). ECG showed no new findings. Differential diagnostic considerations were myocarditis (despite low infectious parameters), coronary artery spasm (despite calcium channel blocker use) or an inherited cardiomyopathy. Her medication was adjusted, with the cessation of all prior medication, and the initiation of a beta blocker and an angiotensin-converting enzyme inhibitor. An ILR was implanted, after which the patient was discharged. Multi-gene panel testing revealed a pathogenic variant (class 5) c.1904-2A > G in the *DSP* gene with potential RNA splicing effect. During follow-up the ILR detected non-sustained ventricular tachycardia, which led to a primary prevention implantable cardioverter-defibrillator (ICD) implantation. Cascade testing revealed the same *DSP *variant in her father known with DCM.

## Discussion

*DSP *is a structural intracellular protein, which is critical in the assembly of the anchoring desmosomes within myocardial and epidermal skin cells [3]. These desmosomes function as part of the cellular adhesion junction, providing rigidity and strength to these cells. Pathogenic variants in the *DSP* gene are associated with ACM and DCM. The recurrent episodes of acute myocardial injury (acute chest pain, elevated cardiac enzymes and no coronary artery disease), as described in both patients, occurs in about one fifth of *DSP* cardiomyopathy patients and is associated with left ventricular LGE, even in patients with normal LVEF [4–6]. This acute clinical presentation of *DSP* cardiomyopathy may be misdiagnosed as an infectious myocarditis, or diagnosed as a MINOCA. Non-infectious myocarditis is often described in *DSP*-related ACM [4, 7, 8]. Sen-Chowdry et al. considered that myocarditis may be an element of the natural progression of ACM, where instead of having an infectious cause, it is due to a genetic predisposition [9]. The exact pathophysiology underlying the myocardial injury is unclear. Recurrent myocarditis-like episodes may be seen, typified by cardiomyocyte damage and subsequent lymphocyte infiltration and necrosis, thereafter followed by repair and fibrofatty tissue replacement, as found in post-mortem analyses [10].

Acute myocardial injury is therefore a common presentation of patients with *DSP* cardiomyopathy, which may present recurrently. Besides the differential diagnosis of infectious myocarditis, MINOCA may also be considered in these patients. Indeed, the SMINC‑2 trial found that CMR diagnosed a small subset of MINOCA patients with primary DCM [11]. In addition, CMR has been shown to diagnose ACM and DCM in patients with initially suspected myocarditis [12]. CMR should therefore be part of the diagnostic work-up in all patients with unexplained acute myocardial injury without coronary artery disease [11]. Cine imaging and LGE in *DSP* cardiomyopathy can show left and/or right ventricular involvement, and specific attention should be paid to fibrofatty replacement; its presence makes T1 mapping challenging [13, 14]. T2 values may be elevated, indicating oedema. LGE was found in almost half of patients with *DSP* cardiomyopathies and can be extensive, particularly in the sub-epicardium with patterns similar as those seen in myocarditis. Ring-like patterns of LGE, as found in both patients, are often observed in ACM and are associated with an increased risk of ventricular arrhythmias [15, 16]. Pathogenic variants in filamin C, another cause of left-dominant ACM, can also give this ring-like LGE pattern [17]. However, presentation with acute myocardial injury in patients with an ACM phenotype seems particular to *DSP* cardiomyopathy.

The review by Smith et al. showed that the clinical phenotype of *DSP* cardiomyopathy presents as a predominantly left ventricular cardiomyopathy, whereas right ventricular involvement was only found in 14% of patients [5]. Additionally, extra-cardiac features have been described in patients with pathogenic *DSP* variants, namely woolly hair and palmoplantar keratoderma. Rare examples of *DSP*-related syndromes are the autosomal-recessive Naxos disease with those extra-cardiac findings and right ventricular involvement; and Carvajal syndrome with the same findings and left ventricular involvement [18, 19]. This left ventricular dominant phenotype, since called left-dominant ACM, differentiates it from traditional arrhythmogenic right ventricular cardiomyopathy (ARVC). This is illustrated by poor correlation with the Task Force Criteria for ARVC, which were mainly developed for patients with a predominant right ventricular phenotype [6]. In reported cohorts of *DSP* cardiomyopathy, females are overrepresented, particularly in patients presenting with acute myocardial injury. In the case series of Smith et al., 69% of 107 *DSP* cardiomyopathy patients were female, but disease progression and burden did not significantly differ between sexes [5]. In addition, an all-female case series by Scheel et al. described 10 patients with a pathogenic *DSP* variant presenting with a myocarditis-like phenotype [4]. The authors hypothesised that the myocardial injury could be the result of immune-mediated effects. Indeed, it was found that in desmosomal ARVC a disproportionate amount of anti-cardiac antibodies are present in comparison with other cardiac pathologies [20]. Nevertheless, the authors did acknowledge that there may be gender disparities in healthcare accessing or biases that were not accounted for.

In general, decreased LVEF is correlated with a higher risk for all-cause and cardiac mortality as well as heart failure-related adverse outcomes in patients [21, 22]. The development of heart failure is described in 38% of patients with a *DSP *cardiomyopathy and is associated with acute myocardial injury and proband status [6]. Furthermore, previous case series show that pathogenic *DSP* variants correlate with ventricular arrhythmias and that left ventricular dysfunction (LVEF < 55%) is strongly associated with sustained ventricular arrhythmias in *DSP* cardiomyopathies [5, 23]. During follow-up, a quarter of the patients with *DSP* cardiomyopathy experience sustained ventricular arrhythmia or sudden cardiac death, even in the absence of overt left ventricular dysfunction, which makes risk stratification for primary ICD implantation challenging in these patients [6, 24]. However, patients with left-dominant phenotypes showed worse survival to severe arrhythmias [5].

Whilst diagnosis by imaging remains challenging, DSP cardiomyopathies present a suggestive LGE pattern which may aid distinguishing it from infectious myocarditis when presented in combination with acute myocardial injury. [25, 26]. Ultimately, if an inherited cardiomyopathy is suspected, genetic testing is necessary. If a pathogenic variant is found, cascade genetic testing should be offered to family members.

## Conclusion

*DSP *cardiomyopathy mainly affects the left ventricle and can be associated with acute myo-inflammatory episodes of chest pain with elevated cardiac enzymes in the absence of coronary artery disease. It should be a differential diagnostic consideration in any patient with unexplained myocardial injury mimicking myocarditis, particularly in the case of recurrent episodes. CMR and genetic testing are key in its evaluation and diagnosis, which may not be missed due to the increased risk of ventricular arrhythmias.

## Supplementary Information

Supplementary Video S1 Video of patient A of 4‑chamber cine images.

Supplementary Video S2 Video of patient B of 4‑chamber cine images.
